# Case report: Unusual cause of refractory hypoxemia after pacemaker lead extraction

**DOI:** 10.3389/fcvm.2023.1237595

**Published:** 2023-08-14

**Authors:** Jingliang Zhou, Jinshan He, Jiangbo Duan, Xuebin Li

**Affiliations:** Department of Cardiac Electrophysiology, Peking University People's Hospital, Beijing, China

**Keywords:** hypoxemia, transvenous lead extraction, patent foramen ovale, pacemaker, transthoracic contrast echocardiography

## Abstract

A 59-year-old woman with a history of a pacemaker implanted for III-degree atrioventricular block was admitted due to pocket infection. The atrial and ventricular leads were removed via the right femoral vein using a needle's eye snare. Hypoxemia was observed immediately after the removal of the lead. It was refractory to oxygen therapy. The pulse oxygen saturation (SpO2) showed 89% in the supine position and 77% in the upright position. However, the CTPA and pulmonary perfusion SPECT/CT imaging did not reveal any signs of pulmonary embolism. Pulmonary function tests and chest CT showed normal results. Transthoracic contrast echocardiography revealed a patent foramen ovale (PFO) and a right-to-left intracardiac shunt, no significant tricuspid regurgitation, without any signs of elevated right heart pressure or pulmonary hypertension. Hypoxemia was considered to be associated with the right-to-left shunt through PFO. The condition was relieved by percutaneous closure of the PFO. Refractory hypoxemia resulting from an intracardiac right-to-left shunt following pacemaker lead extraction is a rare but serious complication. Transthoracic contrast echocardiography helps in diagnosis. If the right-to-left intracardiac shunt through PFO persists irreversibly and the associated hypoxemic symptoms are significant, closure of the PFO is necessary. Transesophageal echocardiography also revealed the presence of a left-to-right shunt through PFO during cardiac systole. The closure of the PFO is also necessary to avoid long-term complications, such as chronic pulmonary hypertension and right heart failure.

## Introduction

Cardiovascular Implantable Electronic Device (CIED) infection is a common indication for CIED lead extraction. Pulmonary embolism is one of the complications related to the lead extraction procedure (1). Despite the low incidence of clinical pulmonary embolism, lead-related thrombosis is not uncommon (2). In cases of refractory hypoxemia following pacemaker lead extraction, we consider the possibility of pulmonary embolism (3, 4). For patients with a patent foramen ovale (PFO), paradoxical embolism is a common complication that can lead to acute cerebral infarction caused by lead vegetations (5, 6). To the best of our knowledge, a right-to-left shunt typically occurs when there is an increase in right atrial pressure (7–9). However, a right-to-left shunt may occurs without elevated right heart pressure (10–13). We report a case of refractory hypoxemia following lead extraction, caused by a “stretched” PFO resulting in a right-to-left shunt, without elevated right heart pressure, significant tricuspid regurgitation, or pulmonary hypertension. It is a rare occurrence that has not been frequently reported. In the condition of refractory hypoxemia after transvenous lead extraction, it is crucial to consider the possibility of a right-to-left intracardiac shunt through a “stretched” PFO, even if preoperative transthoracic echocardiography did not detect the PFO. Insufficient awareness of this issue could delay postoperative recovery. Furthermore, the presence of a PFO with a left-to-right shunt may lead to long-term complications, such as chronic pulmonary hypertension and right heart failure.

## Case presentation

**Figure 1:**
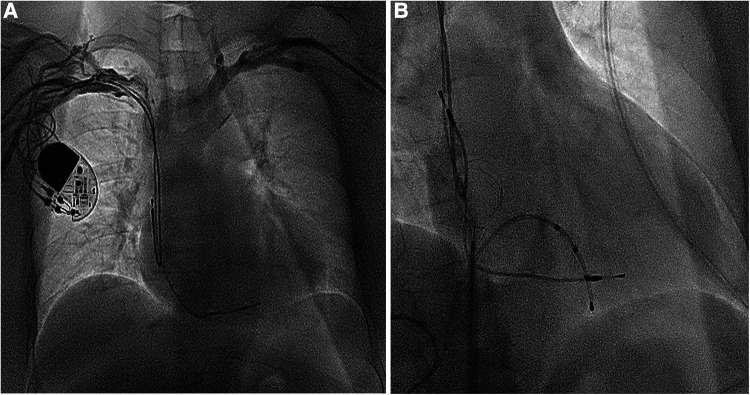
(**A**) The dual-chamber pacemaker implanted for III degree atrioventricular block 14 years ago; (**B**) the atrial and ventricular leads removed using a needle's eye snare through the inferior vena cava.

**Figure 2:**
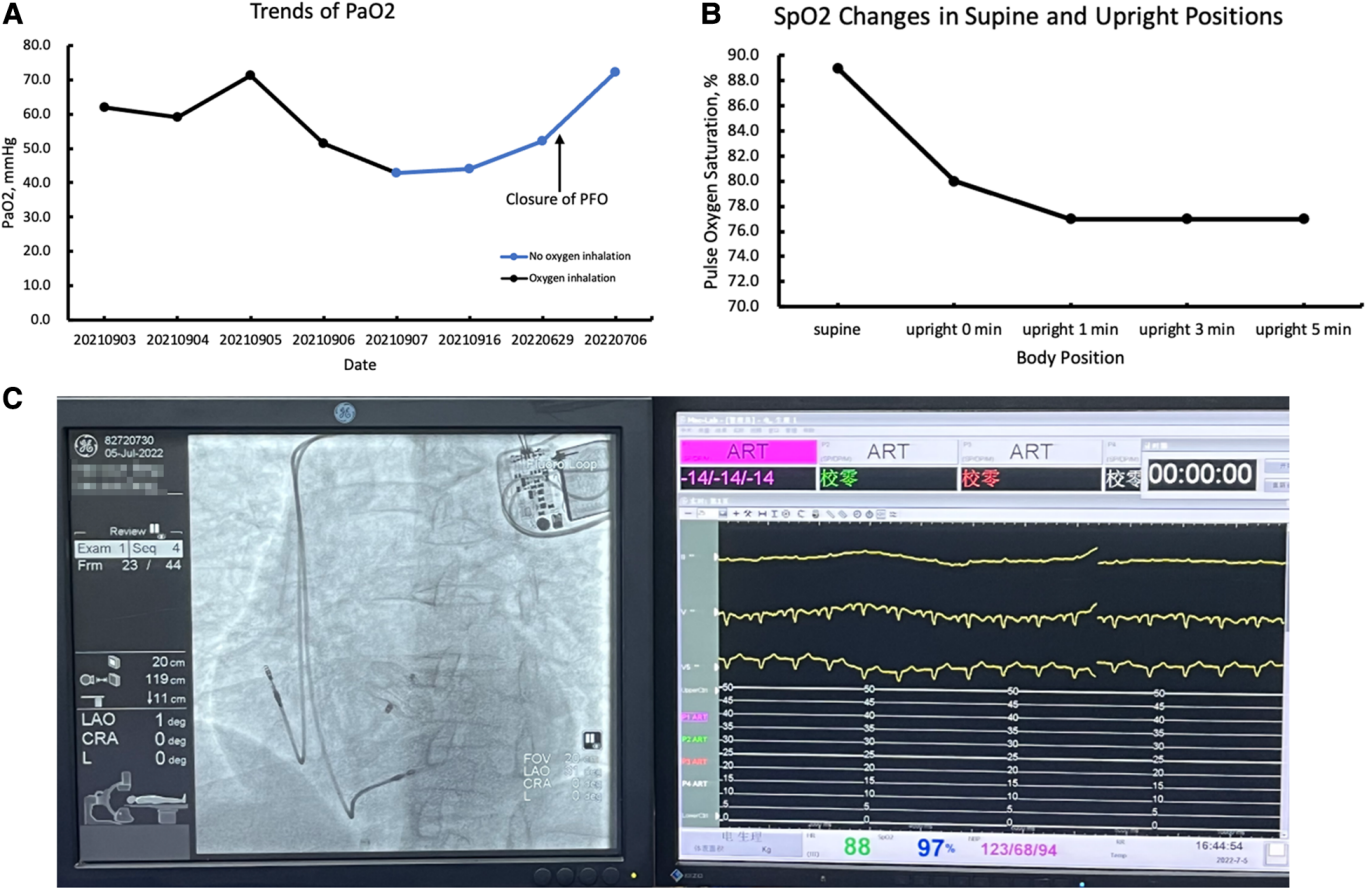
(**A**) Trends in partial pressure of oxygen (PaO2); (**B**) the pulse oxygen saturation (SpO2) changes in supine and upright positions; (**C**) the SPO2 after the closure of PFO.

**Figure 3:**
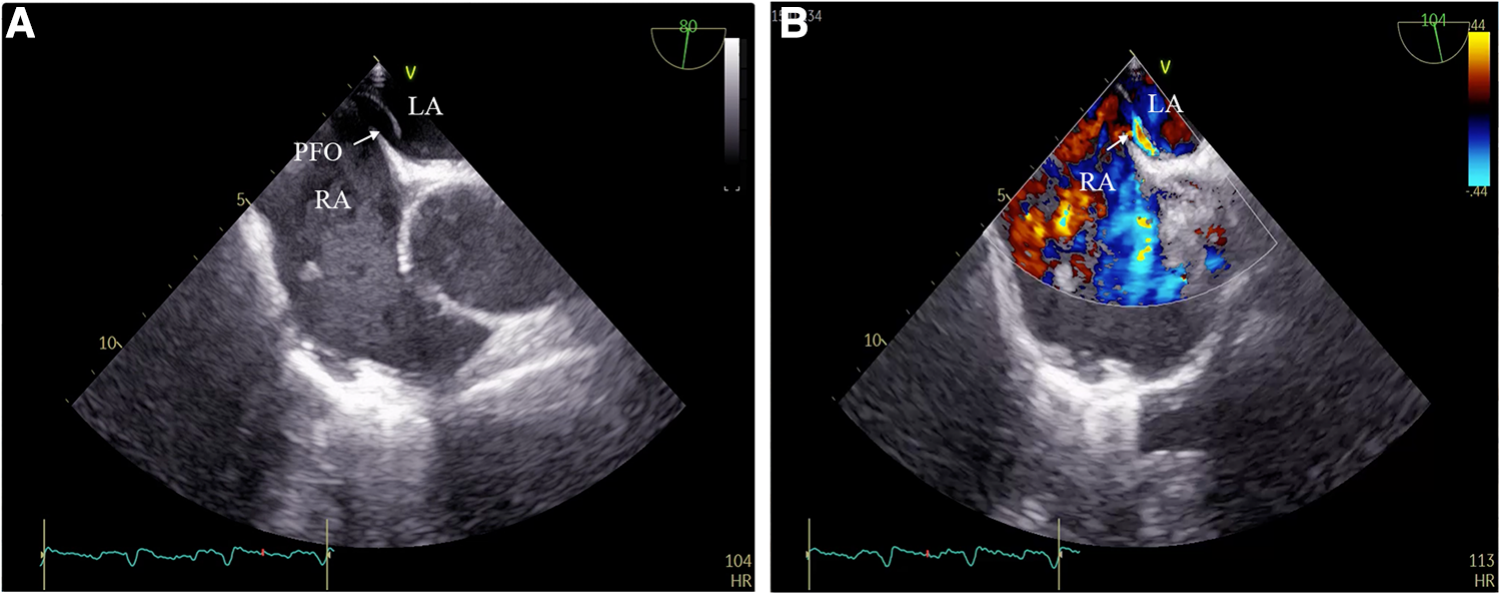
(**A**) PFO detected by transesophageal echocardiography; (**B**) right-to-left intracardiac shunt through PFO.

**Figure 4:**
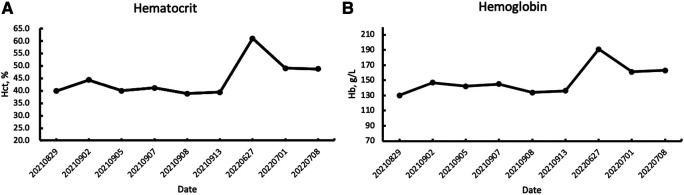
(**A**) The increase in hematocrit during the follow-up period; (**B**) the increase in hemoglobin concentration during the follow-up period.

A 59-year-old woman was admitted due to pacemaker pocket infection. A dual-chamber pacemaker was implanted 14 years ago due to III degree atrioventricular block. No history of infiltrative cardiomyopathy, myocarditis, or coronary artery disease. There was also no history of cardiac surgery or connective tissue disease. The pacemaker pocket infection lasted for two months and was unresponsive to antibiotic therapy. There were no vegetations on the leads. Additionally, the blood cultures were negative. Indications for lead extraction were confirmed, and the patient informed consent was obtained. The atrial and ventricular leads were removed through the right femoral vein using a needle's eye snare (Figure 1). Hypoxemia (FiO2 61%/PaO2 64.3 mmHg) was observed immediately after the removal of the lead. Pericardial effusion was ruled out by x-ray fluoroscopy, and the pulmonary arteriogram did not show any filling defects in the main pulmonary arteries. The computed tomography pulmonary angiogram (CTPA) did not show any signs of pulmonary embolism, and the chest CT did not reveal any significant lesions. As peripheral pulmonary embolism could not be ruled out completely, anticoagulation therapy was initiated. Neither oxygen therapy with a mask (oxygen flow rate of 7.7 L/min) nor noninvasive mechanical ventilation (BiPAP: IPAP = 11 cmH2O, EPAP = 5 cmH2O, FiO2 = 100%) was effective in relieving hypoxemia. Pulse oxygen saturation (SpO2) was measured in both supine and upright positions. The results showed 89% in the supine position and 77% in the upright position (Figure 2). Therefore, we considered the diagnosis of Platypnea-Orthodeoxia. A PFO with intracardiac shunt was suspected. Transesophageal echocardiography revealed a PFO (see Supplementary material: Video 1 and 2), and right heart contrast echocardiography showed the presence of a right-to-left intracardiac shunt, without any signs of pulmonary hypertension or elevated pressure in the right heart (Figure 3). As the patient was pacemaker-dependent, a dual-chamber permanent pacemaker was re-implanted. After re-implantation, the patient's SPO2 levels were monitored at 82%–89% without oxygen inhalation. Although the patient was hypoxemic, her hemodynamic and metabolic parameters were normal. After 10 months of follow-up, the patient's hypoxemia persisted, with SPO2 levels at 88% (PaO2 52.3 mmHg) without oxygen inhalation (Figure 2). The patient also experienced with a decrease in exercise tolerance compared to her preoperative state, and reported shortness of breath when walking 100 meters or climbing one floor. Prior to the lead extraction, the patient was able to tolerate climbing two floors. During the 10-month follow-up period, there was a significant increase in hematocrit and hemoglobin concentration (Figure 4). Pulmonary function tests, repeat chest CT, and pulmonary perfusion SPECT/CT imaging all showed normal results. Repeat transthoracic contrast echocardiography revealed the presence of a PFO with a right-to-left intracardiac shunt. Right cardiac catheterization confirmed that there were no signs of pulmonary hypertension or elevated pressure in the right heart. Hypoxemia was considered to be associated with a right-to-left shunt through PFO. Therefore, percutaneous transcatheter closure of the PFO was performed (see Supplementary material: Video 3). After the closure of PFO, the symptoms of hypoxemia were relieved, and the SPO2 without oxygen inhalation increased to 97% (PaO2 72.2 mmHg). Additionally, exercise tolerance returned to the preoperative state.

## Discussion

Refractory hypoxemia resulting from an intracardiac right-to-left shunt following pacemaker lead extraction is a rare but serious complication. Cases of refractory hypoxemia caused by a right-to-left intracardiac shunt from a “stretched” PFO due to lead extraction maneuver, in the absence of elevated right heart pressure, have been rarely reported. PFO is a common congenital heart disease, the autopsy incidence of PFO is about 27% of the population (14). When investigating the etiology of hypoxemia following a lead extraction procedure, the possibility of a right-to-left shunt should not be overlooked, except for pulmonary embolism. Although negative preoperative echocardiographic findings may suggest the absence of a PFO, it cannot completely rule out the possibility of its presence. Transthoracic contrast echocardiography is considered to be one of the effective non-invasive methods for screening PFO (15, 16). Another possibility is that the previously functionally closed foramen ovale may reopen due to the pulling force exerted during the lead extraction procedure. A variety of etiologic factors can cause right-to-left shunting of the PFO, resulting in hypoxemia. There are case reports of significant hypoxemia due to right-to-left shunt induced by CIEDs (17). Four cases were mentioned in the reports. In three cases, hypoxemia was caused by a right-to-left shunt from the PFO, all of the left-to-right shunts were associated with significant tricuspid regurgitation induced by CIEDs (4, 17, 18). The other case, in which hypoxemia occurred several hours after pacemaker implantation, was due to the displacement of the right atrial lead to the left atrium through the PFO. Relief of hypoxemia was achieved by realigning the atrial lead to the right atrium (19). However, the causes of right-to-left shunts mentioned in the literature above are different from the case we reported. In the case we reported, the lead extraction procedure was uneventful. There was no tricuspid regurgitation observed before or after the procedure, and there was no lead displacement during pacemaker re-implantation. Additionally, cases had been reported where refractory hypoxemia was observed in patients with a right-to-left shunt through PFO after pneumonectomy, as well as in patients with right hemi-diaphragm dysfunction, despite the absence of elevated right heart pressure (10, 20). The underlying mechanisms may be caused by mediastinal deviation resulting from pulmonary resection or elevation of the right hemi-diaphragm. The alterations can change the anatomical relationship between the orifice of the vena cava and the interatrial septum, leading to redirection of blood flow and resulting in a right-to-left intracardiac shunt in patents with a PFO (20). Furthermore, cardiac anatomical changes such as the shortening of the distance between the posterior root of the aorta and the posterior wall of the atrium, may cause a shift in the axis of the interatrial septum. This can result in the opening of the PFO, allowing blood flow from the inferior vena cava to the left atrium (11, 12). Therefore, changes in the structural relationship between the interatrial septum and the superior and inferior vena cava are responsible for hypoxemia caused by right-to-left shunting from the PFO. In the case we reported, despite the absence of pneumonectomy, right hemi-diaphragm dysfunction, and echocardiographic findings of structural changes in the aortic root, the atrial lead extraction maneuver may have triggered the aforementioned structural changes.

Based on the combined factors of cardiac anatomy and the characteristics of lead extraction via the inferior vena cava, the underlying mechanism of the case we reported is speculated as follows: Firstly, surgical manipulation may have distorted the cardiac anatomy due to adhesion of the atrial lead to the atrium. Anatomic distortion of the right atrium or atrial septum could lead to redirection of blood flow to the oval fossa region of the atrial septum (21). Additionally, the flap of the foramen ovale consists of the septum secundum, septum primum, and the atrioventricular canal septum (22). A PFO is a potential space or separation between the septum primum and septum secundum (23). Mild intracardiac shunts through PFO can be detected during the cardiac cycle due to transient pressure gradients between the right and left atria, even in the absence of pathological excitation such as pulmonary hypertension (13). The degree of separation between the septum primum and septum secundum, as well as the intensity of the shunt, varies with hemodynamic and respiratory conditions and may be enhanced under specific circumstances of lead extraction (24).

Therefore, the distortion of the anatomy of the right atrial septum caused by lead extraction through the inferior vena cava approach may redirect blood flow from the inferior vena cava to the area of the oval fossa. In the meantime, manually pulling on the atrial lead may cause greater separation between the septum primum and septum secundum, as well as increase the intensity of the shunt in patients with a PFO. This can lead to the development of an intracardiac right-to-left shunt through the PFO, even when right heart pressure is normal. If the right-to-left intracardiac shunt or bidirectional shunt through PFO persists irreversibly and the associated hypoxemic symptoms are significant, closure of the PFO is necessary (4).

## Conclusion

Refractory hypoxemia caused by a right-to-left intracardiac shunt through a PFO following transvenous pacemaker lead extraction is a rare but significant complication. Inadequate recognition of this problem often results in a delay in diagnosis, which can impact postoperative recovery and lead to a diminished quality of life. Therefore, in cases of unexplained hypoxemia following pacemaker lead extraction, especially in instances of refractory hypoxemia that cannot be alleviated by oxygen therapy even when FiO2 is at 100%, it is crucial to consider the possibility of a PFO with right-to-left intracardiac shunt, in addition to common causes such as pulmonary embolism. The possibility of reopening of PFO with right-to-left intracardiac shunts should be considered. In patients who experience significant symptoms of hypoxemia and have a persistent, irreversible right-to-left shunt through a PFO, closure of the PFO may be necessary. Percutaneous transcatheter closure of a PFO is often a viable option.

## Data Availability

The original contributions presented in the study are included in the article/**Supplementary Material**, further inquiries can be directed to the corresponding author.

## Supplementary material

Supplementary Video S1 Video of TEE,

Supplementary Video S2 Video of Doppler flow assessment with TEE.

Supplementary Video S3 Video of PFO closure.
